# The Spinal Cord as Organ of Risk: Assessment for Acute and Subacute Neurological Adverse Effects after Microbeam Radiotherapy in a Rodent Model

**DOI:** 10.3390/cancers15092470

**Published:** 2023-04-26

**Authors:** Felix Jaekel, Jason Paino, Elette Engels, Mitzi Klein, Micah Barnes, Daniel Häusermann, Christopher Hall, Gang Zheng, Hongxin Wang, Guido Hildebrandt, Michael Lerch, Elisabeth Schültke

**Affiliations:** 1 Department of Radiooncology, Rostock University Medical Center, 18059 Rostock, Germany; felix@m20a.de (F.J.);; 2 Centre of Medical Radiation Physics, University of Wollongong, Wollongong 2522, Australia; jrp933@uowmail.edu.au (J.P.); elette@uow.edu.au (E.E.); mlerch@uow.edu.au (M.L.); 3 Australian Synchrotron, ANSTO, Clayton 3168, Australia; mitzik@ansto.gov.au (M.K.); micah.barnes@petermac.org (M.B.); danielh@ansto.gov.au (D.H.); christoh@ansto.gov.au (C.H.); 4 Monash Biomedical Imaging, Clayton 3168, Australia; gang.zheng1@monash.edu (G.Z.);

**Keywords:** microbeam radiotherapy (MRT), spinal cord as organ of risk, toxicity study, dosimetry, electrophysiology, MRI imaging, motor function, sensibility

## Abstract

**Simple Summary:**

Organs which receive an irradiation dose because they are located in the vicinity of the irradiation target are considered organs of risk. Before a new irradiation technique is tested in clinical trials, it is important to make an assessment of the potential adverse effects in these organs of risk. Microbeam radiotherapy is an innovative radiotherapy technique suitable to control tumours which are considered radioresistant with conventional, already clinically established irradiation techniques. In a small animal model, we have conducted a risk assessment in the thoracic spinal cord as organ of risk during microbeam irradiation in the thoracic cavity and determined the upper dose limit beyond which neurological signs of temporary or permanent damage occur.

**Abstract:**

Microbeam radiotherapy (MRT), a high dose rate radiotherapy technique using spatial dose fractionation at the micrometre range, has shown a high therapeutic efficacy in vivo in different tumour entities, including lung cancer. We have conducted a toxicity study for the spinal cord as organ of risk during irradiation of a target in the thoracic cavity. In young adult rats, the lower thoracic spinal cord was irradiated over a length of 2 cm with an array of quasi-parallel microbeams of 50 µm width, spaced at a centre-to-centre distance of 400 µm, with MRT peak doses up to 800 Gy. No acute or subacute adverse effects were observed within the first week after irradiation up to MRT peak doses of 400 Gy. No significant differences were seen between irradiated animals and non-irradiated controls in motor function and sensitivity, open field test and somatosensory evoked potentials (SSEP). After irradiation with MRT peak doses of 450–800 Gy, dose-dependent neurologic signs occurred. Provided that long-term studies do not reveal significant morbidity due to late toxicity, an MRT dose of 400 Gy can be considered safe for the spinal cord in the tested beam geometry and field size.

## 1. Introduction

The assessment of potential temporary or permanent functional damage to organs of risk (OaR) is an important prerequisite before proposing a new irradiation technique for clinical trial. Microbeam radiotherapy (MRT) is a high dose rate irradiation technique using spatial dose fractionation at the micrometre range. An array of highly collimated, quasi-parallel microbeams is generated, resulting in an inhomogeneous dose distribution of high MRT peak doses and low valley doses between the paths of the microbeams [1]. A good therapeutic efficacy even in malignant tumours which are extremely difficult to control with conventional, already clinically established radiotherapy techniques has been shown in small animal models of malignant brain tumour [2,3,4,5] and lung carcinoma [6]. At the same time, the preservation of normal tissue function was excellent even after irradiation with high MRT peak doses [6,7,8]. The high flux needed to provide the high dose rates of several hundred Gy/s required for MRT can currently only be obtained at synchrotron facilities. However, the construction of synchrotron-independent equipment which would allow to transfer MRT into the clinical environment is already under way.

The proof-of-principle that MRT is feasible as well as therapeutically efficacious also in spontaneous malignant brain tumours (as opposed to the induced tumours in small animal models) and in a tumour much more similar in size and depth from surface, two parameters extremely important in the therapy planning conducted by the medical physicist, has recently been provided in a canine MRT study [9].

In the first two decades of MRT research, studies were focused almost exclusively on the therapy of malignant brain tumours and the response of normal brain tissue to microbeam irradiation. More recent research in vivo has also moved lung carcinoma into focus as a potential treatment target [6]. So far, only ex vivo studies have been conducted to assess potential adverse effects in OaR with an irradiation target in the thoracic cavity, in the explanted beating heart [10,11] and the oesophagus during MRT [12].

Since the spinal cord is an important OaR with any irradiation target in the thoracic cavity, we have designed the first internationally in vivo study to assess spinal cord function in the acute and subacute phase after microbeam irradiation, conducted a toxicity study and determined an MRT peak dose of 400 Gy as the upper dose limit above which acute neurologic adverse effects occur.

## 2. Materials and Methods

### 2.1. Small Animal Study

Thirty-two healthy Wistar rats (240–300 g) were used for this experiment. Of those, 8 animals were used in the dose finding study with MRT peak doses of 450 Gy, 500 Gy, 600 Gy and 800 Gy (*n* = 2 per group). The remaining 24 animals were included in the treatment effects study, using MRT peak doses of 40 Gy (*n* = 8) and 400 Gy (*n* = 8). The remaining 8 animals served as non-irradiated controls. The animals were housed and cared for in a temperature-regulated animal facility exposed to a 12-h light/dark cycle. The experiment was conducted according to the Australian regulations on animal care and experimentation, approved under permit number AS 2019_009. Beyond daily observation to detect obvious signs of neurologic dysfunction after MRT, a series of functional tests was conducted to assess motor strength and hind paw sensitivity. Electrophysiological testing was conducted to obtain somatosensory evoked potentials (SSEPs). All tests were conducted before and 24 h and 6 days after irradiation.

### 2.2. Dose Calculation, Dose Measurements and Simulation

Reference dosimetry was performed using a PinPoint ionization chamber (IC) (PTW 31014, Freiburg, Germany) calibrated to a traceable standard as per the protocol outlined in previous publications [13,14,15]. The sensitive volume of the IC is too large to resolve microbeam structures and therefore was used to calibrate the high-resolution X-Tream dosimetry system under identical reference conditions (20 × 20 mm^2^ broad beam field at 20 mm depth in an RMI-457 Gammex Solid Water^®^ phantom (Gammex-RMI, Middleton, WI, USA)). The X-Tream dosimetry system was then used to calibrate Geant4 Monte Carlo simulations under microbeam irradiation conditions (20 × 20 mm^2^) in a 20 × 20 mm^2^ microbeam field at 12.5 mm depth in a homogenous Solid Water^®^ phantom with dimensions approximating that of the target rats (25 × 25 × 55 mm^3^). The calibrated Geant4 simulations were used to prescribe treatment parameters to achieve the desired dose during treatments.

### 2.3. Microbeam Irradiation Setup

The irradiation study was conducted at the Imaging and Biomedical Beamline (IMBL) of the Australian Synchrotron. The mean spectrum of the energy was 92.9 keV and the storage ring current was 200 mA. An MRT peak dose of 400 Gy, delivered in an array of quasi-parallel microbeams where the individual microbeam width is 50 µm and the centre-to-centre distance is 400 µm, has been shown to result in a superior ablation of highly malignant glial tumours in a small animal model, compared to broad beam irradiation equating the valley dose used in this MRT geometry, while this was not the case if the MRT valley dose was lower than the broad beam dose [5]. In an in vivo study of normal lung tissue, where the target was covered with an array of quasi-parallel microbeams of 50 µm width and a centre-to-centre spacing of 400 µm delivered in unidirectional mode, no acute adverse effects were observed at MRT peak doses of 400 Gy [16]. Thus, an MRT peak dose of 400 Gy was chosen for this study, the results of which would be interesting both for the treatment of intramedullary spinal malignancies and to assess spinal cord toxicity in cases where the spinal cord is considered an organ at risk during irradiation of an extra-spinal irradiation target in the thoracic cavity, for instance, central lung carcinoma. The irradiation was conducted with the animals in an upright position, in posterior-to-anterior direction, in a 2 × 2 cm target centred around four vertebrae of the lower thoracic spine.

### 2.4. Neurobehavioural Testing

#### 2.4.1. BBB Scores Assessing Hind Limb Movement and Trunk Stability

The BBB scoring system was originally developed by Basso, Beatty and Bresnahan for the assessment of hind limb movement in spinal cord trauma studies [17]. A full score of 21 denotes full strength, trunk stability with consistent plantar stepping is observed, the paws being mostly placed parallel to the body, whereas a score of zero describes the complete absence of hind limb movement. Each hind limb is scored independently. Scores were awarded following 2 min observation of free movement in a plane and empty test field.

#### 2.4.2. Rotarod Performance Test to Assess Balance, Coordination and Endurance

On two subsequent days before irradiation, the animals were trained to perform on a rotarod (Ugo Basile, Gemonio, Italy). The time the animals managed to stay on the rotating axis with the speed set to 10 rpm was recorded automatically. Each animal was tested three times in short succession on each of the two days preceding the day of the irradiation, then 24 h and 6 days after irradiation. If an animal still performed on the rotating axis after 120 s, it was removed by the experimenter and the maximum achievement score was recorded. No further rotarod testing was conducted on the same day for this animal once the maximum achievement score had been recorded.

#### 2.4.3. Hind Paw Sensitivity Assessment with Von Frey Filaments

Paw withdrawal after touch to the plantar surface of the hind limb was measured as a parameter of sensitivity. Calibrated von Frey filaments (Ugo Basile) were used to assess whether microbeam irradiation caused sensitivity changes in the pads of the hind limbs. The animals were allowed to move freely across a raised platform consisting of a perforated mesh. The centre of the hind paw was touched with the end of a filament threaded through the mesh from below (Supplementary Figure S1). Starting with a 26 g equivalent filament up to 300 g filaments. Response to higher weight numbers (i.e., stiffer filaments with larger filament diameters) corresponds to lower sensitivity, as the numbers denote the weight required to slightly bend the filament. The weight equivalent of the thinnest (softest) filament that elicited a withdrawal reaction was recorded.

### 2.5. Electrophysiology

Somatosensory evoked potentials (SSEP) are an objective method to assess the velocity of impulse conduction between the point of stimulation and the cerebral cortex [18]. When obtaining SSEP, the somatosensory fraction of a peripheral nerve is stimulated repeatedly while the activity of the corresponding primary somatosensory cortex is recorded in analogy to an electroencephalogram (EEG). By averaging multiple sequential recordings, the evoked potential is distinguished from the background of the EEG waves.

**Figure 1:**
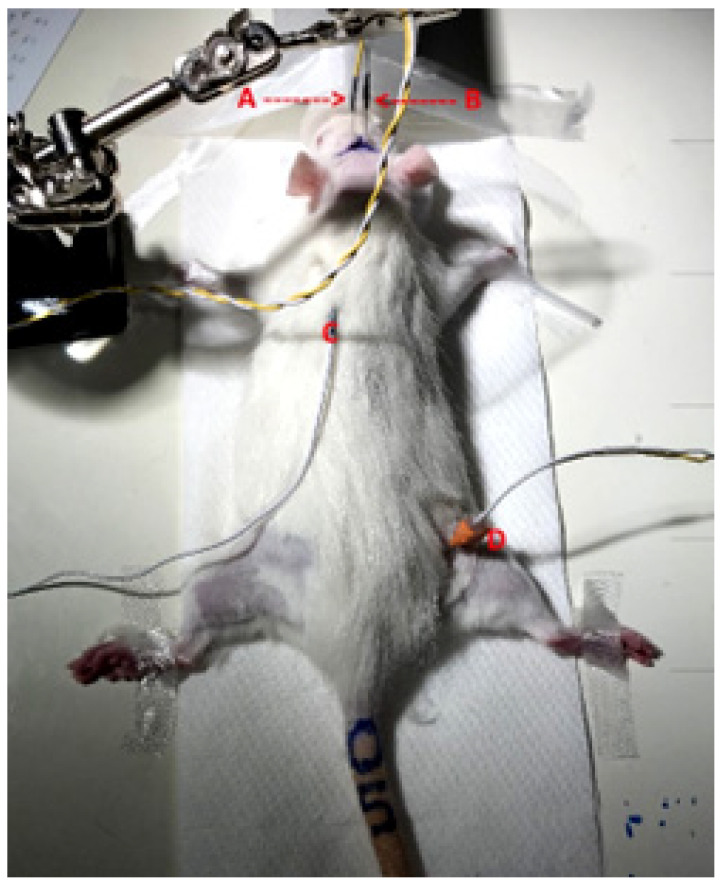
Setup for the acquisition of SSEP in rat sciatic nerve. Active electrode (**A**) and reference electrode (**B**), grounding electrode (**C**) and bipolar stimulating needle electrode in the popliteal fossa of the right hind limb (**D**).

In this study, SSEP were recorded before and after irradiation to determine the remaining conductivity of the nerval pathways through the irradiated spinal cord. We chose the sciatic nerve of the hindlimb as it is rather big in diameter, easily accessible and the ascending somatosensory fibres would pass the irradiated area of the thoracic spinal cord. The nerve was stimulated just above the popliteal fossa with a bipolar needle electrode (Figure 1). The bipolar needle electrode was a custom-made inhouse production, consisting of a pair of Ambu^®^ Neuroline subdermal needle electrodes (diameter 0.4 mm, article number 746 12-100/1/20) arranged at a 3.0 mm distance. Electrophysiological measurements were conducted using a two-channel G:NEO EMG/EP device (Computomedics^®^ Xegis, country). A rectangular pulse with an intensity of 2 mA and a repetition frequency of 3.7 Hz was used. The correct stimulation site was verified by obtaining a visible twitching of the ancle joint as a result of the parallel stimulation of the motor components of the nerve.

During this procedure, the animals were under general anaesthesia, the isoflurane in concentrations between 1.4% and 2.3% and a flow rate of 1.0 L/min being administered through a custom-made half open mask fitted over the snout of the rat. The core body temperature was maintained at 37 °C by placing the animals on a heat pad. The cortical signal was acquired by a needle electrode placed 2.0 mm dorsal of the bregma and 1.5 mm from the midline to cover the primary projection field of the sciatic nerve as described by Sakatani [19]. The reference electrode was placed over the contralateral somatosensory cortex, the grounding electrode at the corresponding shoulder blade approx. halfway between stimulation and detection site.

In three acquisition cycles, 200 measurements were collected each from the left and right hind limb of each animal and averaged. Cycles with non-distinguishable P1 signals or not reproducible results were excluded from further data analysis.

### 2.6. MRI Imaging

MRI images were acquired post-mortem in one animal irradiated with MRT peak doses of 800 Gy, using a Bruker BioSpec 9.4T MRI scanner (Bruker BioSpin GmbH, Ettlingen, Germany) with a Bruker 86 mm volume transmitter coil and 4-array rat heart receiver coil at Monash Biomedical Imaging, Monash University. A 2D multi-slice multi-echo (MSME) sequence was used to achieve both the proton density and the T2-weighted imaging. The imaging parameters were as follows: TR = 5000 ms, TE1 = 9 ms, TE2 = 63 ms, FOV = 19.2 × 19.2 mm^2^, image size = 128 × 128, slice thickness = 1 mm, slice number = 35, and imaging in the axial plane.

### 2.7. Statistical Analysis

A non-parametric One-Way ANOVA test (GraphPad Prism 6, GraphPad Software, Inc., La Jolla, CA, USA) was used to assess the statistical significance of the data obtained.

## 3. Results

### 3.1. Dose Calculations, Measurements and Simulations

**Figure 2:**
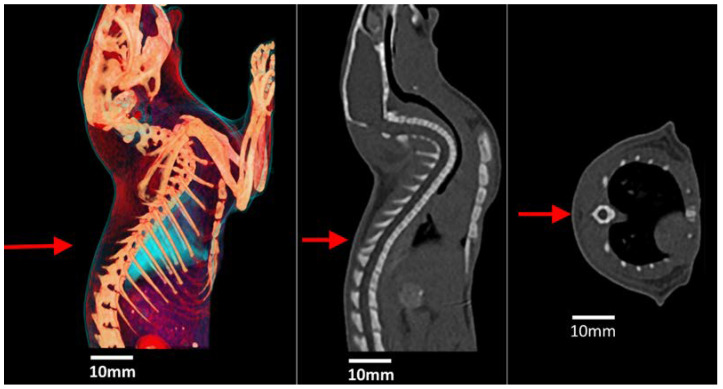
A 3D reconstruction (**left**), sagittal plane (**middle**) and coronal plane (**right**) of a rat indicating direction and approximate location of incident beam (red arrow).

Figure 2 shows a 3D reconstruction and the CT scan of a rat which was used to obtain approximate parameters for the dimensions of the spinal cord, spine, lung and surrounding soft tissue. These materials were then modelled in Geant4 using the anatomical tissues defined in the ICRP Publication 110 [20]. Subsequently, these dimensions and tissue definitions were used to construct a simple cubic phantom in Geant4 for dose calculations.

**Figure 3:**
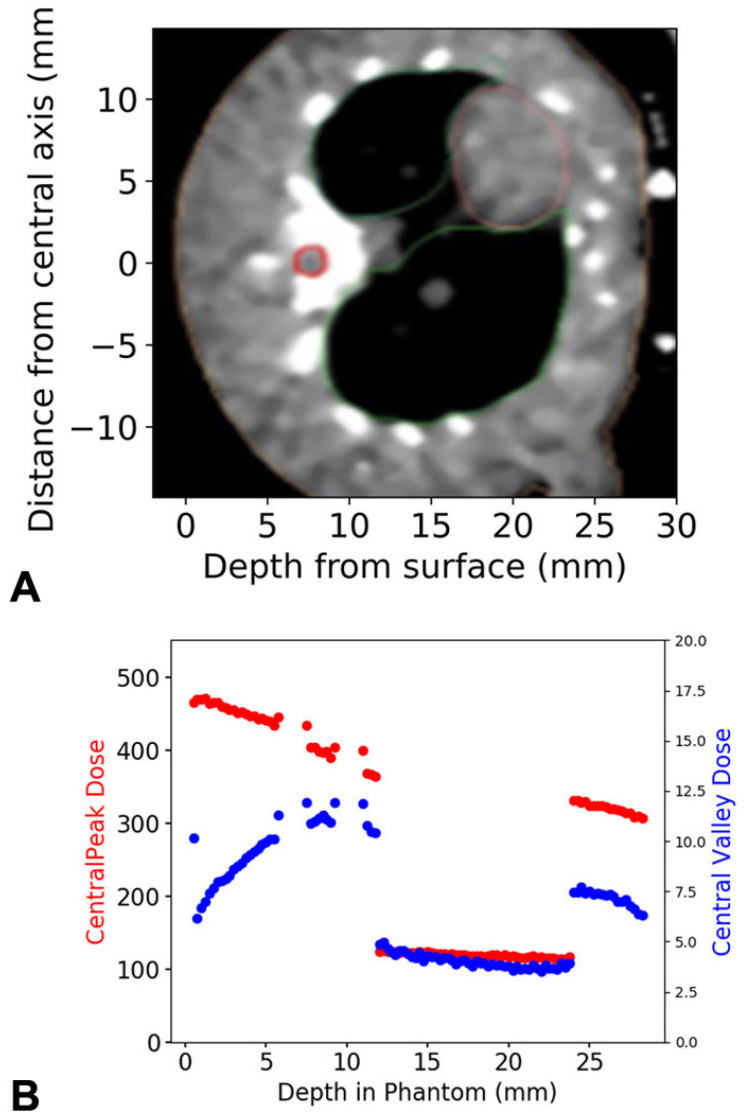
X-ray dose distribution within the tissue. In the axial MRT slice, the horizontal zero-line marks the central axis of the irradiation field in the posterior-to-anterior irradiation procedure. The spinal cord is outlined in red (**A**). Depth dose curve for the central five microbeam peaks and valleys transiting the spinal cord region of interest, located at a depth of 7.5 to 9.25 mm (**B**). The direction of the dose decrease shown in (**B**) corresponds to the increasing depth from the irradiated surface (surface = skin entry of irradiation dose) shown in (**A**). The spinal cord is neighboured by two regions of spine (cortical bone), each 1.5 mm thick. Downstream of the spinal cord (11 to 23.75 mm) is lung tissue. All other regions are soft tissue. The dose distribution is depth-dependent as well as tissue-specific.

Figure 3 shows the microbeam peak and valley depth dose profile with a prescription dose of 400.0 ± 5.1 Gy in the peak to the spinal region. Irradiation was performed with a microbeam peak dose rate of 672.5 ± 8.5 Gy/s in this region.

The irradiation dose calculated over the central 2 × 2 mm field, containing the spinal cord bordered by bone, is considered the integrated dose. The MRT peak doses, their correlated valley doses and the integrated doses are shown in Table 1.

### 3.2. Neurologic Signs and Acute Adverse Effects—Toxicity Study

No symptomatic pathology was seen after irradiation with MRT peak doses of 40 Gy and 400 Gy, with associated valley doses of 1.3 Gy and 13.3 Gy, respectively. Both peak doses were chosen as treatment doses for this study, referring to a pilot study conducted in this dose range in the lungs of rats [16].

Both of the animals irradiated with microbeam peak doses of 450 Gy developed reversible neurologic symptoms. Approx. six hours after irradiation, they showed signs of pain associated with flexor spasms in both hind limbs. Both animals received an intraperitoneal injection of 0.4 mg dexamethasone in addition to the pain killer buprenorphine (0.05 mg). One of the animals started to walk again within less than one hour. It received a BBB score of 21/21 and completed the rotarod testing 48 h after irradiation with full score. The second animal developed paraplegia within an hour but responded favourably to a second dose of dexamethasone. The BBB score slowly improved to 20/21 at 48 h after irradiation. The animal received a full rotarod score and a BBB score of 21/21 three days after irradiation.

The animals irradiated with microbeam peak doses of 800 Gy, 600 Gy and 500 Gy all developed severe neurologic pathology and required euthanasia. The severity and the latency of the pathologic symptoms was dose-dependent. All animals had initially recovered from anaesthesia and started to move without obvious neurological deficits inside their cages within 30 min after irradiation.

Within the first six hours after irradiation, there were no obvious neurologic deficits, and the BBB score was 21/21 for both animals. Then, extensor spasms in the hind limbs were observed. The animals improved after a first dose of 0.3 mg dexamethasone. Three hours later, no more spasms were observed. The animals showed no signs of distress but were unable to support their weight on their hind limbs. They made sweeping movements with both hind limbs, receiving a BBB score of 8/21 and a second dose of 0.3 mg dexamethasone each. Seven hours later, the BBB score had not improved, and the decision was taken to euthanize the animals.

Complete paraplegia with painful extensor spasms was seen in the hind limbs following a very fast onset (<30 min) within 2.5 h after irradiation. No improvement was achieved with intraperitoneal injections of dexamethasone and 0.05 mg Buprenorphine intraperitoneally, and the decision was taken to euthanize the animals. At the Melbourne Biomedical Imaging Institute (MBI), post-mortem MRI images were acquired in an animal that had received a microbeam peak dose of 800 Gy, using a Bruker small animal scanner (Bruker BioSpin GmbH, Ettlingen, Germany). An extensive radiogenic edema was detected in the irradiated spinal cord section as the cause of the neurological pathology. The edema is most likely to have triggered a combination of spinal and neurogenic shock, a state of complex autonomic dysregulation which is known to occur in human patients after spinal cord injury (in this case, a radiation injury). It is associated with a high percentage of neurologic deficits and death in human patients too. It has been stated that there is no equivalent to the acute radiogenic syndrome seen in the CNS after high irradiation doses, such as nausea, disorientation and loss of consciousness [21]. However, our observation might challenge this statement. Dysreflexia might well be recognized as the equivalent of acute radiogenic syndrome in the spinal cord.

Based on these results, the maximum MRT peak dose administered in this beam geometry should be 400 Gy.

### 3.3. Motor and Sensitivity Testing of Animals Treated with MRT Peak Doses of 40 Gy and 400 Gy

#### 3.3.1. Rotarod: Assessment of Motor Abilities and Endurance

The BBB scores were 21/21 (i.e., full score) in both treatment groups at all times, without exceptions. During the rotarod training with 32 healthy animals before irradiation, 16 animals (50%) did not manage to stay on the rotating axis for 120 s on the first training day and 6 animals (18.75%) failed on the second training day. At 24 h after irradiation, all 24 animals in the treatment and control groups passed with a full score (120 s). This can be attributed to the learning effect and the lack of deterioration of motor control and endurance in the irradiated animals.

Of the remaining animals at 3 days after irradiation (half of the animals had been euthanized at 24 h after irradiation), one of the animals irradiated with an MRT peak dose of 40 Gy scored 99/120 s, one of the animals irradiated with an MRT peak dose of 400 Gy scored 115/120 s and one of the non-irradiated control animals scored 102/120 s. Although this amounts to 25% in each group, the numbers are far too small to allow a solid statistical statement. Considering also that one of the non-irradiated animals failed, it should not be weighed too heavily, especially since they all managed to stay on the rotating axis for more than two-thirds of the expected time. In summary, no motor deficits were detected after irradiation with microbeam peak doses of either 40 Gy or 400 Gy.

#### 3.3.2. Von Frey Filaments: Sensitivity Assessment of the Hind Paws

A natural variation in the withdrawal response across two filament increments was observed in non-irradiated animals. The response to the sensitivity testing using the von Frey filaments on the plantar surface of the hind limbs varied between 26 g (2/32), 60 g (22/32) and 100 g filaments (8/32).

In the treatment study, 6/24 animals showed a sensitivity loss across one increment: 2/8 animals irradiated with microbeam peak doses of 40 Gy and 4/8 animals irradiated with peak doses of 400 Gy at 24 h after irradiation. None of the animals were found with sensitivity loss of more than one increment at this time. At 6 days after irradiation, six animals were found with sensitivity loss of one increment: 2/4 animals after irradiation with 40 Gy and 1/4 animals irradiated with 400 Gy. Surprisingly, two non-irradiated animals were found to have a slightly decreased sensitivity response. Three animals were found to have decreased sensitivity over two increments: one animal irradiated with 40 Gy and one animal irradiated with 400 Gy.

### 3.4. SSEP

**Figure 4:**
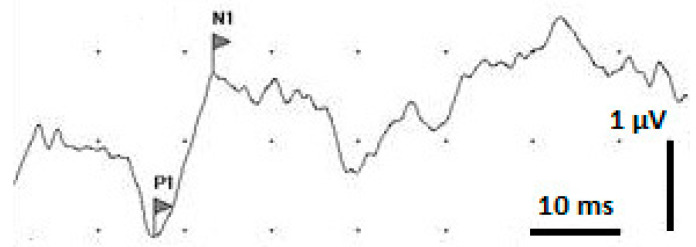
Example of an SSEP signal after averaging 200 measuring cycles. P1 is marked as the first positive spike. N1 is marked as the first negative spike. The difference between P1 and N1 is the resulting amplitude.

The detected SSEP (example shown in Figure 4) showed a positive peak labelled P1 approx. 15 to 20 milliseconds after sciatic nerve stimulation, followed by a negative peak labelled N1, at about 20 to 30 milliseconds after stimulation. The time lag between P1 and N1 would be the resulting amplitude. The N1 signal and the SSEP amplitude varied widely between the different animals, but also between the pre- and post-irradiation measurements in the same animal. This might be attributed to the anaesthesia affecting individual animals differently and also affecting the same animal differently during repeated anaesthesia procedures. It is known that Isoflurane can cause a decrease in the SSEP amplitude and change of waveform, especially in the later parts of the signal [22]. Therefore, our analysis was focused on P1, the first signal of the cortical response, as the marker for the somatosensory impulse conduction time through the myelon.

**Figure 5:**
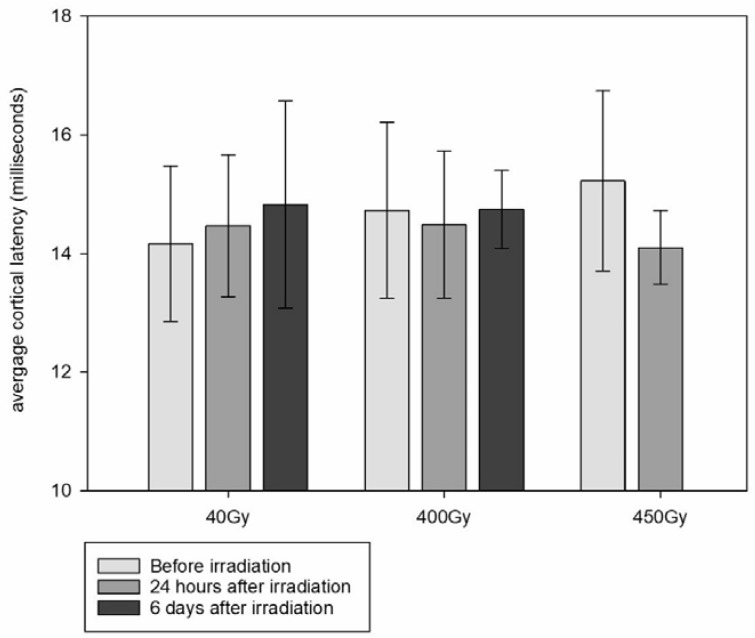
Cortical P1 latency average, obtained in animals irradiated with MRT peak doses of 40 Gy, 400 Gy and 450 Gy before and after irradiation (error bars show SD). No data are available for the animals irradiated with 450 Gy after irradiation.

To compensate for the individual differences between the animals and for the lack of sufficient reference data for rat sciatic nerve SSEP in the literature, we compared the cortical latency times of the individual animals before and after the MRT of the spinal cord (Figure 5). There was no significant prolongation of the cortical signal between animals irradiated with MRT peak doses of either 40 Gy or 400 Gy and the non-irradiated controls.

### 3.5. Post-Mortem MRI

**Figure 6:**
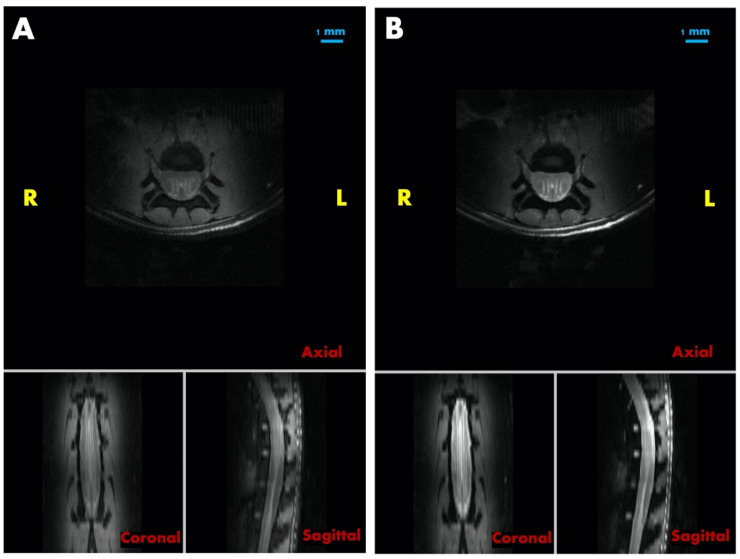
The proton density and T2-weighted MR images of a rat irradiated with MRT peak doses of 800 Gy (**A**). Proton density images in three orientations (**B**) T2-weighted images in three orientations.

High signal intensities were observed in the spinal cord of an animal irradiated with MRT peak doses of 800 Gy in both the proton density (Figure 6A) and T2-weighted images (Figure 6B), indicating that acute edema was developing after irradiation with high peak doses. In particular, these high signal intensity regions were shaped in four clearly visible lines running in anterior–posterior direction, two of each laterally on each side of the central canal of the spinal cord. Parallel to these features were several only partially visible lines, one of them tracing precisely through the level of the central canal in anterior–posterior direction. The distance between each of two lines was 0.4 mm, which is in keeping with the centre-to-centre distance of 400 µm in this irradiation geometry. This observation supports the assumption that MRT peak doses as high as 800 Gy can cause significant acute edema in the spinal cord.

## 4. Discussion

The normal spinal cord tissue is dose-limiting in the irradiation of spinal cord tumours as well as in its organ of risk function during therapeutic irradiation of a target in the thoracic cavity. While the well-documented radiation-induced myelopathy is rather a late toxicity after spinal cord exposure to ionizing irradiation, becoming symptomatic weeks to months after irradiation [23], temporary neurologic dysfunction and pain flares are characteristic for the acute phase, the latter especially after spine stereotactic body radiotherapy (SBRT) [24].

In clinical radiotherapy, cumulative spinal cord doses above 100 Gy are frequently accumulated during re-irradiation for spinal metastases [25,26]. Although the MRT peak doses in our in vivo study were up to eight times higher, one should consider that the MRT peak doses comprise only approx. 1/8 of the target area and that the MRT valley doses were far below these values. Essentially, one can consider the MRT peak doses like a simultaneously integrated boost, generating zones of high biologically effective doses. Only in the animals irradiated with MRT peak doses of 800 Gy was the integrated spinal cord dose above 100 Gy. The acute symptoms might have been prevented or at least drastically reduced in severity with a prophylactic administration of dexamethasone.

Up to MRT peak doses of 400 Gy, no neurologic adverse effects and no signs of pain were registered. No electrophysiological evidence of cortical delay as a sign of impaired impulse conduction along the somatosensory components of the spinal cord was registered in the observation period of 6 days after irradiation. These results were consistent with the clinical and behavioural observations. It is known that isoflurane affects nerve conduction times. However, collecting the signal of the first cortical response and by using the lowest isoflurane concentration possible for sufficient sedation, we hope to have compensated for this problem as best as possible.

The symptomatology seen in our animals after administration of MRT peak doses above 450 Gy had both neurological and pain components. In the example of the 800 Gy MRT peak dose, radiogenic edema in the paths of the microbeams was actually demonstrated in the post-mortem MRI. Pain expression was obvious by voicing of the animals.

A pain flare after spine stereotactic body radiotherapy (SBRT) is known in clinical radiotherapy, and the patient is informed about this risk before obtaining informed consent to conduct the procedure. It has been shown previously that 4 mg dexamethasone in human patients is a good rescue strategy in steroid-naive patients with pain flare after spine stereotactic body radiotherapy [27]. This dose roughly equates to the dose administered in our study once neurologic symptoms and pain became apparent with several hours delay after MRT. Moreover, it has been shown in a prospective clinical study that prophylactically administered dexamethasone can significantly reduce the pain flare after spine stereotactic body radiotherapy in human patients [24]. In this study, a prophylactic oral dose of dexamethasone administered 1 h before irradiation followed by a second dose in the morning of the day after SBRT reduced the experienced pain flare to 19%, compared to 68% of patients who did not receive dexamethasone. In the scenario of our in vivo study, the dexamethasone can be expected to act both anti-edematous and as pain relief.

Neurological signs and pain occurring in our animals after irradiation with MRT peak doses of 450 Gy were easily reversible with one or two doses of post-irradiation dexamethasone, respectively. We hypothesize that the cause for the neurologic signs was an acute radiogenic edema, which might have been prevented altogether by administration of dexamethasone before MRT, similar to what is done in current clinical practice before radiosurgery. Considering the fast reversal of both neurological signs and pain expression after dexamethasone administration, we would expect that, if this study was repeated, neither neurologic signs nor pain would be seen after irradiation with MRT peak doses of 450 Gy after a prophylactic bolus of dexamethasone.

Extrapolating the results of this in vivo study to human patients, one should keep in mind that there is a one order of magnitude difference between the diameters of the spinal cords in rats and adult human patients. As a result, a 50 µm wide microbeam would cause morphological damage in a much smaller volume percentage of each substructure of the human spinal cord, compared to the spinal cord of a rat, where the axial diameter of the entire cord is roughly 2–3 mm wide.

## 5. Conclusions

In an in vivo rodent model and with the microbeam geometry used, a microbeam dose of 400 Gy is to be considered the upper dose limit beyond which acute dose-dependent neurological adverse effects occur. This dose limit might be increased after a prophylactic dose of dexamethasone, like that customarily administered before clinically established radiosurgery.

Since radiation myelopathy, which carries a risk of significant morbidity as well as mortality, has been described after clinical radiotherapy where the spinal cord was included in the treatment field [24], the next important step is a long-term study to assess the extent of late toxicity in the spinal cord after MRT.

## Figures and Tables

**Table 1:** MRT peak doses, correlated valley doses and integrated doses.

MRT Peak Dose	MRT Valley Dose	Dose Integrated over The Spinal Cord
400 Gy	13.3 Gy	65.6 Gy
500 Gy	16.9 Gy	82.0 Gy
600 Gy	20.0 Gy	98.4 Gy
800 Gy	26.6 Gy	131.2 Gy

## Data Availability

Data are available upon request from the corresponding author (E.S.).

## Supplementary Materials

The following supporting information can be downloaded at: https://www.mdpi.com/article/10.3390/cancers15092470/s1, Figure S1: Sensibility testing with von Frey filaments.
