# Supplementary material for: The Spinal Cord as Organ of Risk: Assessment for Acute and Subacute Neurological Adverse Effects after Microbeam Radiotherapy in a Rodent Model
Source: Cancers (Basel). 2023 Apr 26;15(9):2470. doi: 10.3390/cancers15092470 (PMC10177263; doi:10.3390/cancers15092470)
Supplement: Supplementary file 1 [file cancers-15-02470-s001.zip › cancers-2299396-supplementary.pdf]

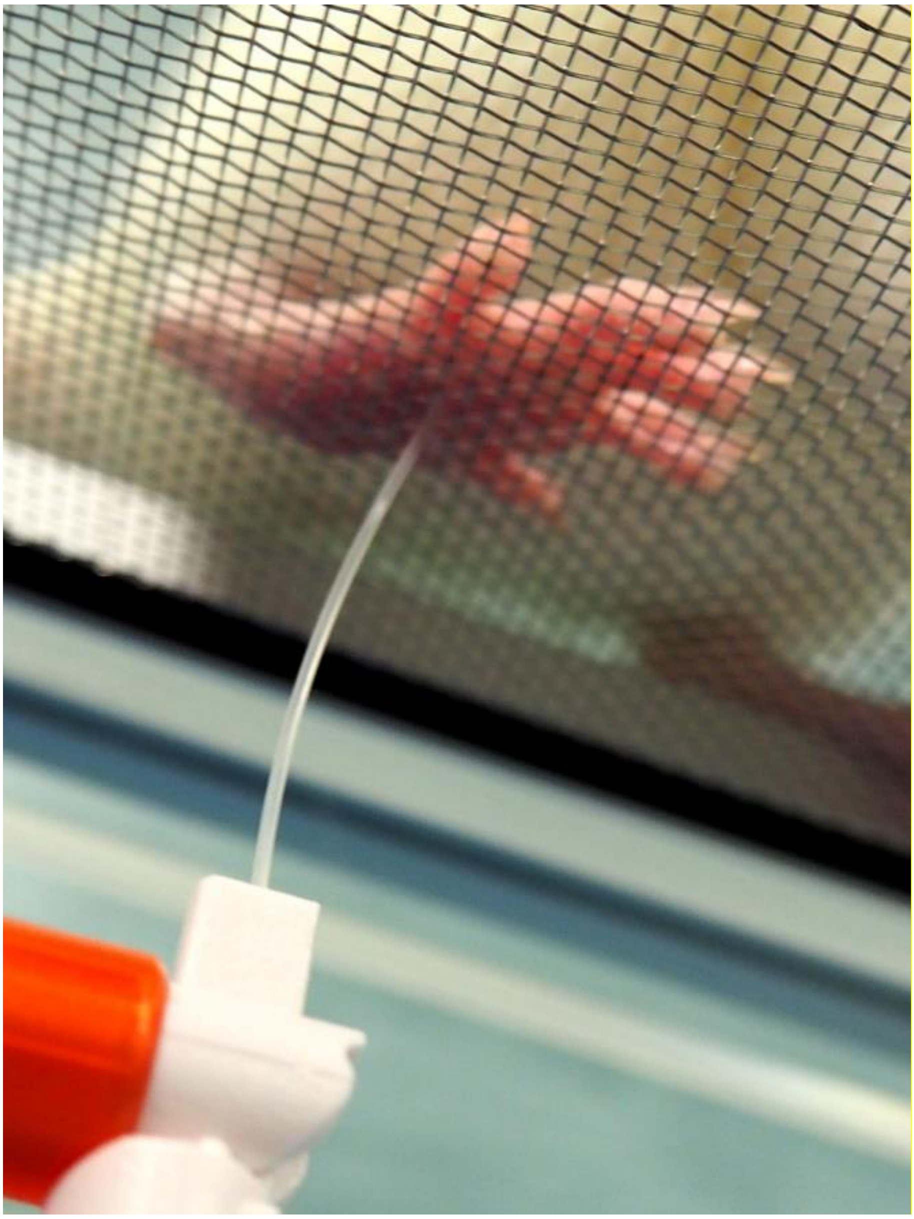

Figure S1. Sensibility testing with von Frey filaments. The filament is applied to the pad of the rodent hind paw. The softest filament eliciting a paw withdrawal response is scored.
